# Supplementary material for: Giant thermoelectric power factor in ultrathin FeSe superconductor
Source: Nat Commun. 2019 Feb 18;10:825. doi: 10.1038/s41467-019-08784-z (PMC6379375; doi:10.1038/s41467-019-08784-z)
Supplement: Supplementary file 1 — Supplementary Information [file 41467_2019_8784_MOESM1_ESM.pdf]

Supplementary Information for "Giant thermoelectric power factor in ultrathin FeSe superconductor"

Shimizu et al.

Supplementary Figures

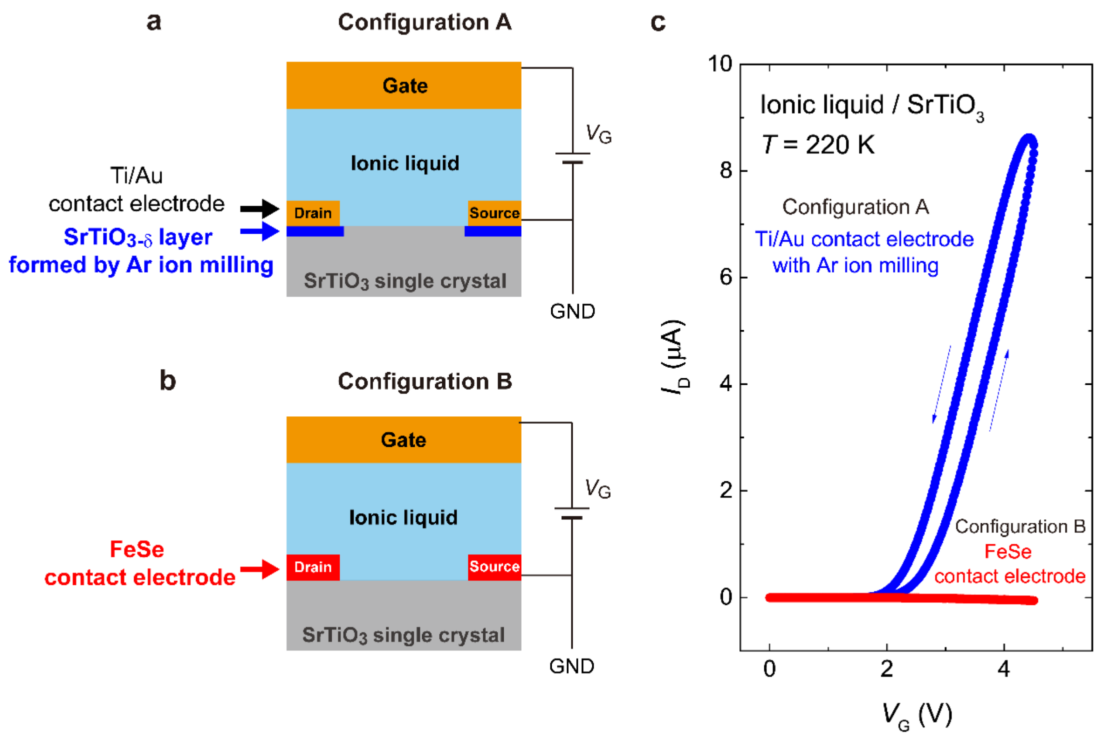

d

$\rho_{2D,STO}$ (Ω) [experiment]	α_{STO} (μV/K) [experiment]	α_{total} (μV/K) [Eq. S1]
1.7 M	-812	3.3
665 k	-734	2.7
318 k	-680	1.7
196 k	-645	0.5
92.1 k	-589	-2.6
47.3 k	-501	-6.6
12.9 k	-434	-27
8.5 k	-404	-39
6.6 k	-363	-44
4.7 k	-303	-51

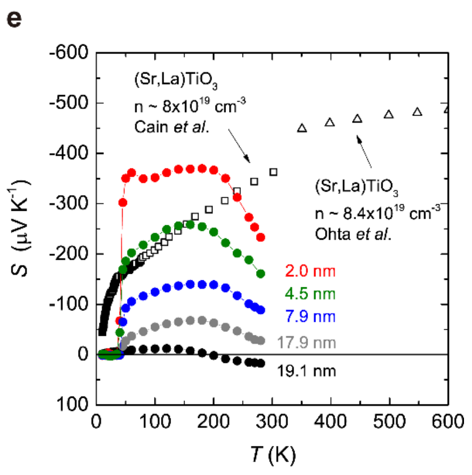

Supplementary Figure 1 Evaluation of contribution of $SrTiO_3$ substrates. a, Conventional configuration of electric double layer transistor based on $SrTiO_3$. The bule area of $SrTiO_3$ just beneath the Ti/Au contact electrodes was exposed to the Ar-ion

milling to form conducting $SrTiO_{3-\delta}$. **b**, Electric double layer transistor structure with FeSe contact electrode. There is no $SrTiO_{3-\delta}$ layer below FeSe. **c**, Transfer characteristics of ion-gated $SrTiO_3$. The FeSe contact electrode completely suppressed the drain-source current I_D. **d**, Estimation of Seebeck effect for virtual parallel conduction. $\rho_{2D,STO}$ and α_{STO} are the experimental values of the two dimensional resistivity and the Seebeck coefficient of an ion-gated $SrTiO_3$ at 200 K[1]. α_{STO} is the total Seebeck coefficient of FeSe and $SrTiO_3$, which was estimated by Eq. S1. **e**, Temperature dependence of Seebeck coefficient in FeSe thin film and chemically-doped $SrTiO_3$. The data of FeSe is the same as that of Fig. 4a in the main text. The data of La-doped $SrTiO_3$ were taken from literature[2,3].

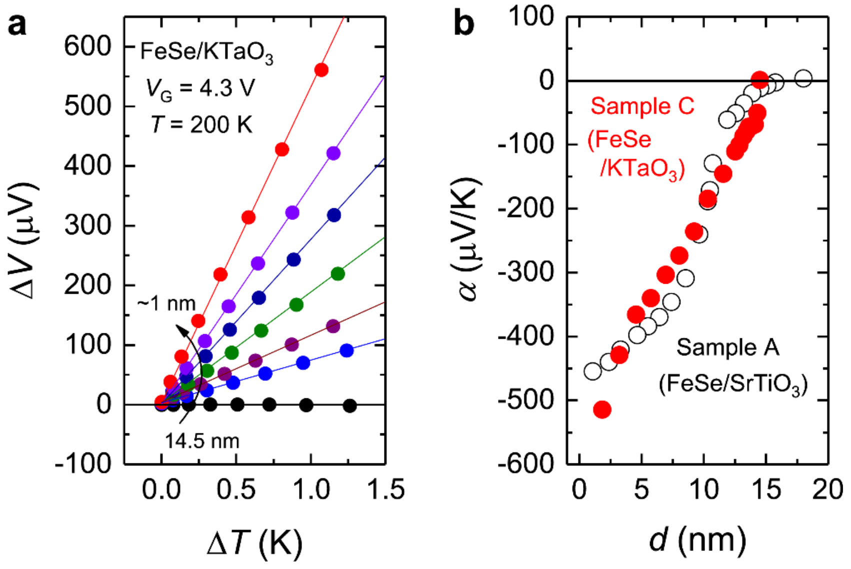

Supplementary Figure 2 Seebeck effect of FeSe/KTaO$_3$. a, Thermoelectric voltage ΔV under temperature difference ΔT in an FeSe thin film on a $KTaO_3$ substrate, Sample C. The thermoelectric measurements were performed at 200 K with V_G = 4.3 V. The

value of ΔV dramatically increased with decreasing thickness d from ~14.5 nm. **b**, Thickness d dependence of the Seebeck coefficient α. The values of α were estimated from the slope of the ΔV - ΔT plot in **a** as $\alpha = -\Delta V / \Delta T$. With reducing the thickness, the absolute value of α was enhanced. The data for Sample A, which is the same with that in Fig. 2b, are also plotted in Supplementary Fig. 2b.

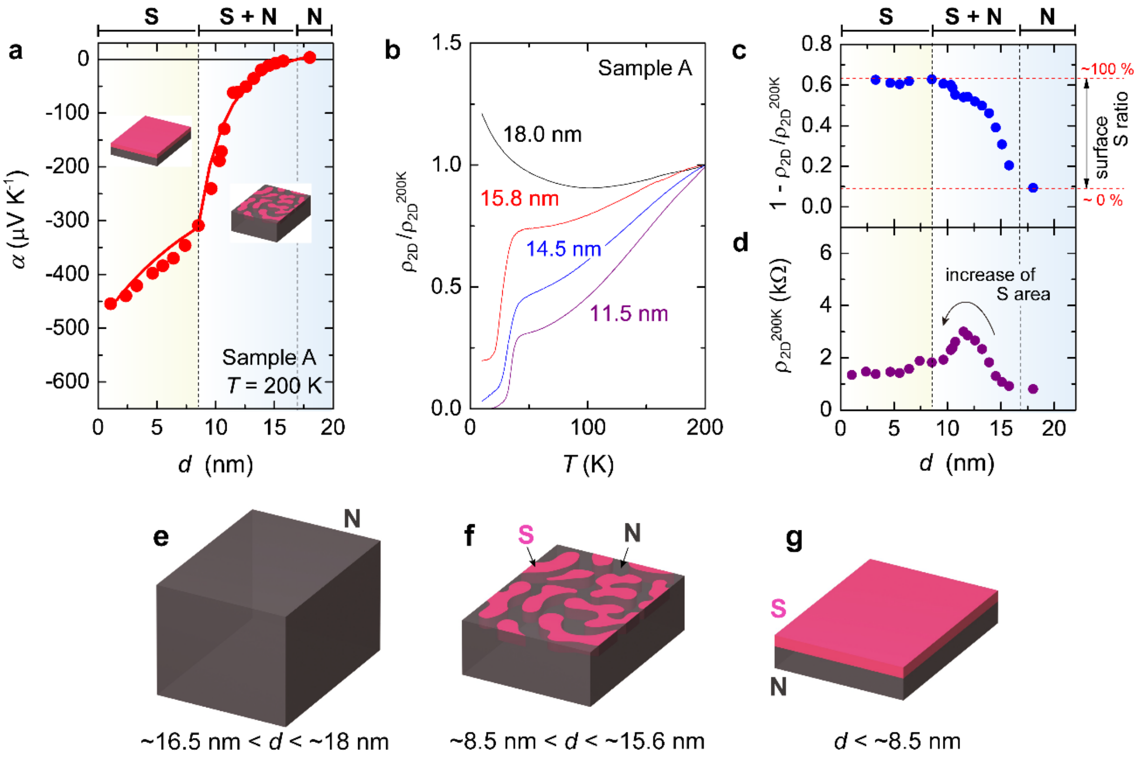

Supplementary Figure 3 Evolution of electronic structure. **a**, Thickness d dependence of Seebeck coefficient α in FeSe thin film, Sample A. The data is the same as those used in Fig. 2b. The thermoelectric measurements were performed at 200 K with V_G = 5 V. The absolute value of α showed a dramatic increase with decreasing d.

The solid line is the result of the simulation. **b**, Temperature dependence of two dimensional resistivity ρ_{2D}. We normalized ρ_{2D} to the value at 200 K, $\rho_{2D}^{200\ K}$. **c**, Development of S phase on FeSe top surface. The value of 1- $\rho_{2D}^{100\ K}$/ $\rho_{2D}^{200\ K}$ increased with decreasing *d* and saturated at *d* ~ 8 nm, reflecting the evolution of the area of the S phase. **d**, Variation of $\rho_{2D}^{200\ K}$ as a function of *d*. **e**,**f**,**g**, Schematic illustrations of phase separation of FeSe surface at V_G = 5 V.

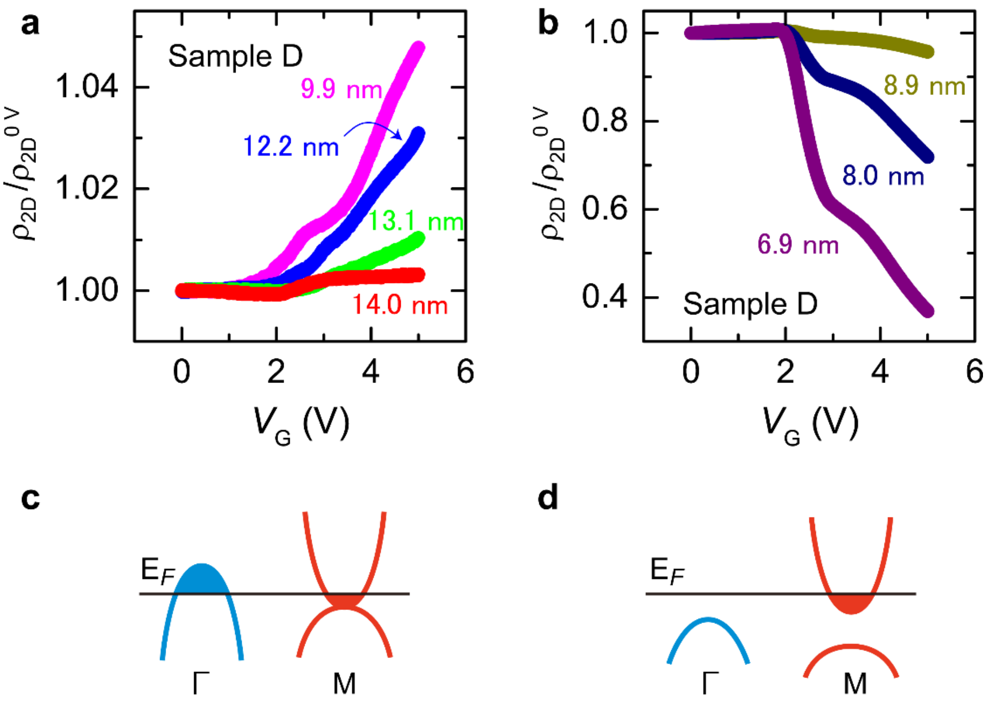

Supplementary Figure 4 Surface carrier doping by electric field effect. a,**b**, Gate voltage V_G dependence of the two dimensional resistivity ρ_{2D} for Sample D. The thickness of the film was tuned by the electrochemical etching technique[4]. Here, ρ_{2D} was normalized to the resistivity ρ_{2D}^{0V} at V_G = 0 V. The bias V_G was swept at 220 K in order to

electrostatically increase the electron carrier density of the top surface of Sample D[5,6]. **c**,**d**, Schematic band structures of FeSe. The field effect carrier doping induces the reconstruction of the band structure from **c** to **d**, especially in the region thinner than ~ 9 nm.

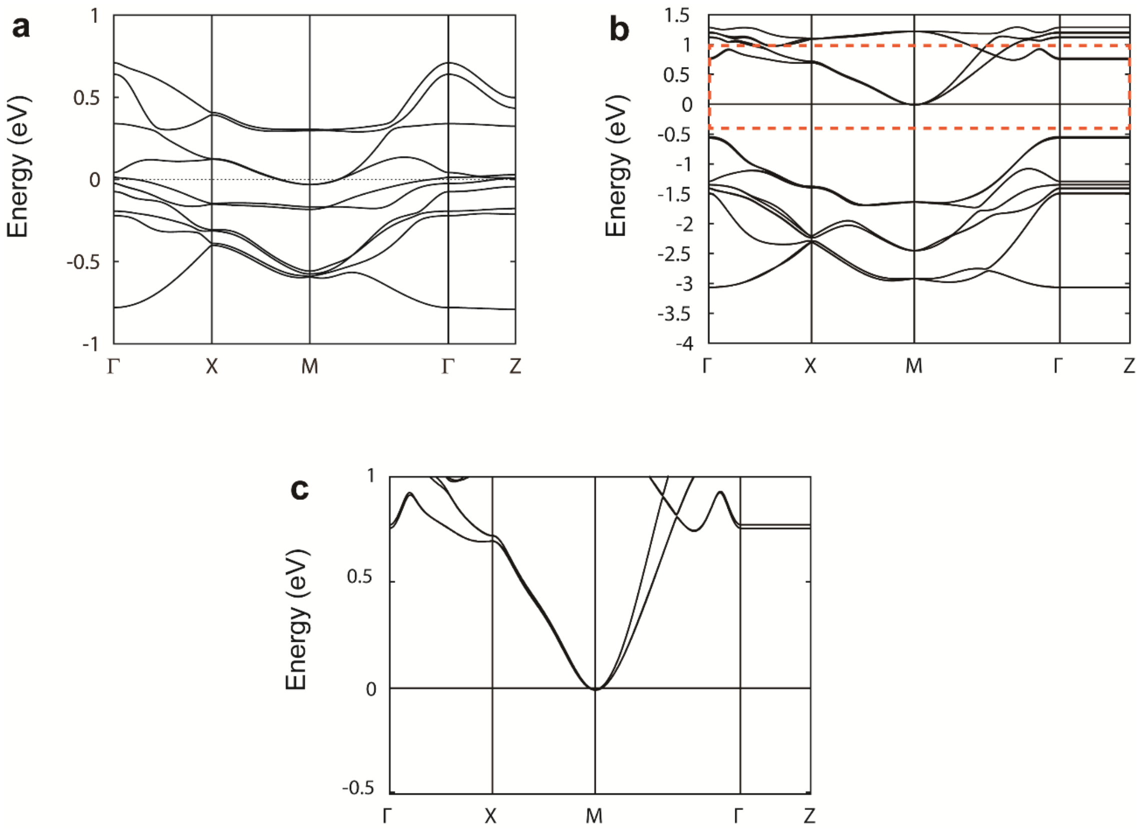

Supplementary Figure 5 Calculated band structures of FeSe. We calculated the band structures of **a** bulk systems and **b** thin films. The enlarged view of area outlined by the dashed lines in **b** is shown in **c**.

Supplementary Tables

Supplementary Table 1 Reference list of thermoelectric materials referred in Fig. 3c. The values of the power factor for Na_xCoO_2[7], $Bi_{1-x}Sb_x$[8], Bi_2Te_3[9], $(Sr,La)TiO_3$[10], $CsBi_4Te_6$[11], Ta_4SiTe_4[12], $FeSb_2$[13], which are plotted in Fig. 3c, were cited from the literatures in this table.

Material	Reference
Na_xCoO_2	M. Lee *et al.*, Nature Mater. 5, 537 (2006).
$Bi_{1-x}Sb_x$	B. Lenoir *et al.*, J. Phys. Chem. Solids 59, 129 (1998).
Bi_2Te_3	V. A. Kulbachinskii *et al.*, J. Solid State Chem. 193, 47 (2012).
$(Sr,La)TiO_3$	J. Fukuyado *et al.*, Phys. Rev. B 85, 075112 (2012).
$CsBi_4Te_6$	D. Chung *et al.*, Science 287, 1024 (2000).
Ta_4SiTe_4	T. Inohara *et al.*, Appl. Phys. Lett. 110, 183901(2017).
$FeSb_2$	H. Takahashi *et al.*, Nature Commun. 7, 12732 (2016).

Supplementary Notes

Supplementary Note 1: Evaluation of parasitic conduction on $SrTiO_3$ surface

Here we provide five pieces of evidence that there is no contribution from the $SrTiO_3$ substrates to the Seebeck effect and the electrical conductivity in FeSe ultrathin films.

It has been reported by many groups that an oxide insulator, $SrTiO_3$, shows metallic conduction with ionic liquid gating[1,14–19]. In our present study, the FeSe thin films were fabricated on $SrTiO_3$ substrates. First of all, therefore, we would like to rule out the possibility that the charge carriers are unintentionally induced at the surface of the $SrTiO_3$ substrates under the application of the gate bias voltage V_G.

We prepared two configurations for the test of the ionic liquid gating on $SrTiO_3$, as shown in Supplementary Fig. 1. Supplementary Fig. 1a is a conventional electric-double-layer transistor configuration (Configuration A) with an insulating $SrTiO_3$ channel and Ti/Au Ohmic contact electrodes. The surface of $SrTiO_3$ just beneath the Ti/Au contact electrodes was exposed to the Ar-ion beam in order to make oxygen deficient $SrTiO_{3-\delta}$ layer[20]. This process forms the Ohmic contact between the Ti/Au contact electrodes and the $SrTiO_3$ channel[1,14–19,21]. Supplementary Fig. 1b is also an electric-double-layer transistor configuration (Configuration B), where FeSe thin films are used as contact electrodes. This configuration mimics a possible hole opening of FeSe films due to the non-uniform electrochemical etching. We measured the transfer characteristics (the drain-source current I_D versus V_G) for the two configurations at 220

K and found strikingly contrasting results. As seen in Supplementary Fig. 1c, I_D in Configuration A increased with increasing V_G, exhibiting a typical *n*-type field effect transistor operation in $SrTiO_3$[1,14–17,19]. On the other hand, I_D in Configuration B did not show any enhancement under the application of V_G. This is due to the formation of the Schottky barrier between FeSe and insulating $SrTiO_3$, which is consistent with the charge transfer picture[22–24]. Thus, when FeSe thin film is gated with ionic liquid, the charge carriers are not induced at the surface of $SrTiO_3$ substrates.

There still remains a possibility that Configuration B in Supplementary Fig. 1b is not an exact copy of the real experiments. Here, we assume that the gate electric field accumulates some amount of charge carriers in the $SrTiO_3$ substrate and that both FeSe and the $SrTiO_3$ substrate contribute to the observed Seebeck coefficient in Fig. 2b in the main text. In this case, they form a parallel circuit and the total Seebeck coefficient α_{total} is written as

$$\alpha_{\mathrm{total}} = \frac{\frac{1}{\rho_{\mathrm{2D,FeSe}}} \times \alpha_{\mathrm{FeSe}} + \frac{1}{\rho_{\mathrm{2D,STO}}} \times \alpha_{\mathrm{STO}}}{\frac{1}{\rho_{\mathrm{2D,FeSe}}} + \frac{1}{\rho_{\mathrm{2D,STO}}}}. \qquad (1)$$

Here, the Seebeck coefficient and the two dimensional electrical resistivity for FeSe are α_{FeSe} and $\rho_{\mathrm{2D,FeSe}}$, and those for the gated $SrTiO_3$ substrate are α_{STO} and $\rho_{\mathrm{2D,STO}}$. We tentatively assume that the Seebeck effect of FeSe is constant at α_{FeSe} = +3.8 μV/K at 200 K (see Figs. 2a and 2b in the main text) against *d*. According to the literatures on FeSe thin flims[4,25–27], the two dimensional resistivity of the monolayer or few-layer FeSe on $SrTiO_3$ substrates, $\rho_{\mathrm{2D,FeSe}}$, is ~ 1 kΩ. Importantly, the Seebeck effect of $SrTiO_3$

single crystals gated with ionic liquid was already reported, the values of $\rho_{2D,STO}$ and α_{STO} at 200 K are known, as summarized in Supplementary Fig. 1d. Substituting the values of $\rho_{2D,FeSe}$, α_{FeSe}, $\rho_{2D,STO}$, and α_{STO} into Supplementary Equation 1, α_{total} is estimated as listed in Supplementary Fig. 1d. The estimated values of α_{total} were -50 μV/K at most even if the $SrTiO_3$ substrate acquired a highly metallic conduction. These considerations clearly exclude the possible parallel conduction of $SrTiO_3$ substrate and FeSe.

Here we assume that, as extreme case, the observed Seebeck coefficient is all attributed to the $SrTiO_3$ substrate. In this case, however, the temperature dependence of the observed Seebeck effect in FeSe on $SrTiO_3$ (Sample B in the main text) is totally different from that in conducting $SrTiO_3$, as shown in Supplementary Fig. 1e. The values of α in FeSe thin films has a peak at around 150 to 200 K, which is typical behavior in Fe-based high-T_c superconductors[28–30], as shown in Fig. 4c; on the other hand, α in conducting $SrTiO_3$ keeps increasing[2,3] up to 1100 K (the data above 600 K is not shown). These results clearly demonstrate that the enhanced Seebeck effect in Fig. 2b stems from the high-T_c phase of FeSe.

The above three arguments rule out the possible electrical conduction of the $SrTiO_3$ substrate and its contribution to the enhanced Seebeck coefficient observed in the FeSe thin film. In this sub-section we show that the observed large thermopower is also irrelevant to the phonon-drag effect that is induced by the phonons in $SrTiO_3$ substrate.

When the thickness of thin films is several nanometers, the interaction between charge carriers in thin films and phonons in insulating substrates could induce the phonon drag effect at low temperatures where the thermal conductivity κ of the substrates is maximum[31]. As for $SrTiO_3$ single crystals, κ shows a sharp peak at around 20 K[32]. However, α of FeSe thin films was zero at ~ 20 K, as shown in Supplementary Fig. 1e, because FeSe thin films become superconducting below ~ 40 K. In addition, the intensity of the phonon drag effect is inversely proportional to the fourth power of temperature, so that it should decay rapidly with increasing temperature and be pronounced only at around the peak temperature of κ of the substrates[31]. Thus, the effect of phonon drag triggered by $SrTiO_3$ substrate is unambiguously excluded.

In addition to above arguments, a direct way to examine the contribution of $SrTiO_3$ substrates for the enhanced Seebeck effect is to measure the Seebeck effect of FeSe thin films fabricated on other substrates. Here we apply the ionic gating on FeSe/$KTaO_3$ to evaluate the thickness dependence of the Seebeck effect at 200 K.

Supplementary Figure 2a shows the thermoelectric voltage ΔV as a function of the temperature difference ΔT between two thermocouples (see Fig. 1c) on a FeSe thin film on a $KTaO_3$ substrate (Sample C) at V_G = 4.3 V. The thermoelectric measurement was done at 200 K to compare with the data for FeSe/$SrTiO_3$ in Figs. 2a and 2b. All the ΔV plots for different d's linearly depended on ΔT, securing the accurate characterization of the Seebeck effect. The values of ΔV were dramatically enhanced with decreasing d, showing that the Seebeck effect of FeSe thin films is actually enhanced by reducing d even on $KTaO_3$ substrates.

Supplementary Figure 2b plots the d dependence of α (= $-\Delta V / \Delta T$) for Sample C at 200 K (red circles). With reducing the thickness from 14.5 nm, the absolute value of α was dramatically enhanced. The data for Sample A of FeSe/$SrTiO_3$, which is identical with that in Fig. 2b, are also plotted in Supplementary Fig. 2b. The values of α for both $KaTaO_3$ and $SrTiO_3$ substrates show similar thickness dependence, suggesting that the enhanced Seebeck effect of FeSe does not depend on substrate materials.

Supplementary Note 2: Evolution of Seebeck effect with decreasing film thickness of FeSe

As reported by ARPES studies[33–36] and discussed in the later section (Supplementary Note 3), the electric field carrier doping performed in this study should cause a modulation of the electronic structure from a semimetallic one to an n-type semiconductor-like one, with the emergence of high-T_c superconductivity. Here, the semimetallic and n-type semiconductor-like band structures are denoted as the N phase and the S phase, respectively, referring to the definition used in previous ARPES studies[34,35].

In this section, we analyse the d dependence of ρ_{2D} and the Seebeck coefficient α in FeSe. The continuous decrease of α of Sample A in Supplementary Fig. 3a, which is the same as Fig. 2b, suggests that the area of the S phase gradually expands with decreasing d, as schematically shown in Supplementary Figs. 3e-3g. Indeed, evidence of the phase separation was observed in the temperature dependence of ρ_{2D} since the N

phase and the S phase have different ground states[34,35,37–39]. Supplementary Figure 3b shows ρ_{2D} at V_G = 5 V normalized to the values at 200 K. When d ~ 18 nm, Sample A was insulating, with an upturn of ρ_{2D} at low temperatures. The superconducting transition occurred at ~ 40 K when d was ~ 15.8 nm, whereas the zero resistance was not observed. This clearly indicates the phase separation of the superconducting S and insulating N regions at intermediate thickness, as schematically shown in Supplementary Fig. 3f. Zero resistance was observed when d was reduced to ~11.5 nm, suggesting that the S regions were connected from one edge of the sample to the other.

The area ratio between the S phase and the N phase on the top surface of Sample A is roughly estimated as follows. As shown in Supplementary Fig. 3b, the thinner FeSe film showed more metallic transport. This means that the S phase, which is dominant in thin regions, has larger metallic conductivity than that of the N phase. Therefore, $1-\rho_{2D}^{100K}/\rho_{2D}^{200K}$, which represents the degree of the metallic transport, indicates the proportion of the S and the N phases on the top surface of FeSe. Supplementary Figure 1c shows the variation of $1-\rho_{2D}^{100K}/\rho_{2D}^{200K}$, which should increase with increasing area of the S phase. Indeed, the value of $1-\rho_{2D}^{100K}/\rho_{2D}^{200K}$ increased from ~0.1 at d ~ 18 nm to ~0.6 below ~8.5 nm. The saturation of $1-\rho_{2D}^{100K}/\rho_{2D}^{200K}$ suggests that the surface of Sample A was mostly covered by the S phase below ~8.5 nm, as schematically shown in Supplementary Fig. 3g. Supplementary Figure 3d shows the d dependence of $\rho_{2D}^{200\ K}$. The value of $\rho_{2D}^{200\ K}$ increased with decreasing d from ~18 nm, decreased below ~11 nm, and became roughly constant below ~8 nm. The reason that the R_s decreased below ~11 nm is that the area of the S phase, which is more conducting than the N phase, expanded with

decreasing d. The fact that $\rho_{2D}^{200\,K}$ stayed constant below ~8 nm suggests that most of the FeSe surface was the S phase below ~8 nm, which is consistent with the analysis of $1- \rho_{2D}^{100K}/ \rho_{2D}^{200K}$ in Supplementary Fig. 3c. The phase separation possibly originated from the thickness fluctuation of the initial condition[4] and of the accumulation layer width for gate-induced carriers.

In order to confirm the discussion above, we performed a simple simulation on the d dependence of α. We consider that the overall volume of Sample A is the N phase and the S phase in the thick and thin limits, respectively. Therefore, α of the pure S phase, α_S, is about −454 μV K^{-1}, and that of the pure N phase, α_N, is about +3.8 μV K^{-1}. Here, the simulation is performed based on a model assuming that the phase separation between the N and the S occurs only on the gated top layer with the thickness d_{EDL} and that the carriers flow in a parallel circuit consisting of the gated top layer (with the two dimensional resistivity $\rho_{2D,T}$ and the Seebeck coefficient α_T) and the ungated bottom layer (with $\rho_{2D,B}$ and α_B). As for the former, the total area of S phase, A_s, monotonically increased with decreasing d from ~ 18 nm to ~ 8.5 nm, as shown in Supplementary Fig. 3c. However, there is no information on the ratio of A_S and the total surface area A for 8.5 nm < d < 18 nm. Therefore, we simply assumed A_S/A as follows

$$\frac{As}{A} = -\frac{d-8.5}{18-8.5} + 1. \qquad (2)$$

By further assuming that the S and N phases are connected only in series, the total Seebeck coefficient α is described as,

$$\alpha = \frac{\rho_{2D,T}\alpha_B + \rho_{2D,B}\alpha_T}{\rho_{2D,B} + \rho_{2D,T}}. \qquad (3)$$

Here, α_T, $\rho_{2D,B}$, and $\rho_{2D,T}$ are described using A_s as follows:

$$\alpha_T = \alpha_S \frac{A_S}{A} + \alpha_N \frac{A_N}{A}$$

$$= \alpha_N + (\alpha_S - \alpha_N)\frac{A_S}{A} \qquad (4)$$

$$\rho_{2D,B} = \frac{\rho_B}{d - d_{EDL}} \qquad (5)$$

$$\rho_{2D,T} = \rho_{2D,S}\frac{A_S}{A} + \rho_{2D,N}\frac{A_N}{A}$$

$$= \rho_{2D,N} + \left(\rho_{2D,S} - \rho_{2D,N}\right)\frac{A_S}{A}$$

$$= \frac{\rho_N}{d_{EDL}} + \left(\frac{\rho_S}{d_{EDL}} - \frac{\rho_N}{d_{EDL}}\right)\frac{A_S}{A} \qquad (6)$$

Here, ρ_B, ρ_S, and ρ_N ($\sim\rho_B$) are the three dimensional resistivity of the ungated bottom layer, the S phase, and the N phase, respectively. By substituting Supplementary Equations 2, 4, 5, and 6 into Supplementary Equation3 with $\alpha_B = \alpha_N$, the d dependence of α is given. We take d_{EDL} ~ 1.5 nm in the simulation, since typical values of d_{EDL} in electric double layer transistors is 1~2 nm[40–43].

We found that the variation of α of Sample A was well-reproduced by the simulation, as shown by the solid curve in Supplementary Fig. 3a, which assures the validity of our model. The value of α shows a steep decrease with decreasing d from 18 nm to ~8.5 nm due to the expansion of the surface S region on the surface. Below d ~ 8.5 nm, α changes weakly but still keeps decreasing because the observed thermoelectric response includes contributions from the gated top surface and the ungated underneath layer. It should be noted that the assumptions on the surface S ratio for 8.5 nm < d < 18 nm (Supplementary Equation 2) and the connection of the S and N phases on the top surface (Supplementary Equation 4) do not affect the result at d < 8.5

nm. If more realistic expressions for Supplementary Equations 2 and 4 were used, we would obtain a minor change in the d dependence of α only for 8.5 nm < d < 18 nm.

Supplementary Note 3: Transfer characteristics of FeSe-based ion-gated transistor

The V_G dependence of ρ_{2D} for a FeSe thin film on a $SrTiO_3$ substrate, Sample D, detected interesting behaviour when a positive V_G was applied. Supplementary Figures 4a and 4b show ρ_{2D} normalized with the resistance ρ_{2D}^{0V} at $V_G = 0$ V. The gate bias V_G was swept at 220 K to electrostatically control the charge carrier density[5,6] on the top surface of the FeSe film. Unexpectedly, the V_G dependence of ρ_{2D}/ρ_{2D}^{0V} considerably changed just by decreasing d. As shown in Supplementary Fig. 4a, ρ_{2D}/ρ_{2D}^{0V} increased with increasing V_G when d was larger than ~ 10 nm, which agrees with the previous studies on FeSe thin films[6] and single crystals[5] with d of ~ 10 nm. This suggests that the dominant transport carriers of the FeSe film at 220 K are holes. When d was smaller than ~ 9 nm, on the other hand, the positive V_G reduced R_s (Supplementary Fig. 4b), suggesting that the dominant carriers in the thinner condition are electrons. According to the recent ARPES studies on FeSe, heavy electron doping into thin layers of FeSe by high-temperature annealing[33–35] or K coating[36] does not simply induce a shift of E_F; the electronic band structure is dramatically modulated, from a semimetallic one schematically shown in Supplementary Fig. 4c to an n-type semiconductor-like one in Supplementary Fig. 4d, with the emergence of high-T_c superconductivity. The V_G dependences of ρ_{2D}/ρ_{2D}^{0V} in Supplementary Fig. 4b and of α in Fig. 2b strongly

support the idea that such an evolution of the band structure is caused by the field effect carrier doping as well (see Supplementary Note 2).

Supplementary Note 4: Calculation of Seebeck coefficient for N and S phases

In order to interpret the large negative Seebeck coefficient observed in the experiment, here we calculate the Seebeck coefficient based on the band structures calculated for bulk FeSe (the N phase) and monolayer FeSe (the S phase). The Seebeck coefficient is given by

$$\alpha = \frac{1}{-eT}\frac{K_1}{K_0} \qquad (9)$$

where we define

$$K_n = \int d\epsilon\, L(\epsilon)(\epsilon - \mu)^n \left(-\frac{\partial f}{\partial \epsilon}\right) \qquad (10)$$

and

$$L(\epsilon) = \sum_{\boldsymbol{k},l} {v_{\boldsymbol{k}x}^{l}}^{2} \left(\frac{\eta}{(\epsilon+\mu-\epsilon_{\boldsymbol{k}}^{l})^2+\eta^2}\right)^2. \qquad (11)$$

Here, μ denotes the chemical potential, f denotes the Fermi distribution function, $\epsilon_{\boldsymbol{k}}^{l}$ is the energy level for orbital l, $v_{\boldsymbol{k}x}^{l} = \frac{\partial}{\partial k_x}\epsilon_{\boldsymbol{k}}^{l}$ is the band velocity, and η is the energy-broadening factor. For bulk FeSe, we employ a modified band dispersion (Supplementary Fig. 5a) so as to match the ARPES results[44]. For monolayer FeSe, we first assume that the system is 2D. Then, in order to estimate the possible maximum

Seebeck coefficient realized in the S phase, we consider the situation that only electron pockets contribute to the Seebeck coefficient, i.e., we use the band structure plotted in Supplementary Figs. 5b and 5c. This band structure is obtained by multiplying the renormalization factor 0.82 to the hopping integrals for d_{zx} and d_{yz} orbitals between nearest neighbour sites and eliminating the hole bands.

Supplementary References

1. Shimizu, S., Ono, S., Hatano, T., Iwasa, Y. & Tokura, Y. Enhanced cryogenic thermopower in $SrTiO_3$ by ionic gating. *Phys. Rev. B* **92,** 165304 (2015).
2. Cain, T. A., Kajdos, A. P. & Stemmer, S. La-doped $SrTiO_3$ films with large cryogenic thermoelectric power factors. *Appl. Phys. Lett.* **102,** 182101 (2013).
3. Ohta, S., Nomura, T., Ohta, H. & Koumoto, K. High-temperature carrier transport and thermoelectric properties of heavily La- or Nb-doped $SrTiO_3$ single crystals. *J. Appl. Phys.* **97,** 034106 (2005).
4. Shiogai, J., Ito, Y., Mitsuhashi, T., Nojima, T. & Tsukazaki, A. Electric-field-induced superconductivity in electrochemically etched ultrathin FeSe films on $SrTiO_3$ and MgO. *Nature Phys.* **12,** 42–46 (2016).
5. Lei, B. *et al.* Evolution of High-Temperature Superconductivity from a Low T_c Phase Tuned by Carrier Concentration in FeSe Thin Flakes. *Phys. Rev. Lett.* **116,** 077002 (2016).
6. Hanzawa, K., Sato, H., Hiramatsu, H., Kamiya, T. & Hosono, H. Electric field-induced superconducting transition of insulating FeSe thin film at 35 K. *Proc. Natl. Acad. Sci. U. S. A.* **113,** 3986–3990 (2016).
7. Lee, M. *et al.* Large enhancement of the thermopower in Na_xCoO_2 at high Na doping. *Nature Mater.* **5,** 537–540 (2006).
8. Lenoir, B., Dauscher, A., Cassart, M., Ravichch, Y. I. & Scherrer, H. Effect of antimony content on the thermoelectric figure of merit of $Bi_{1-x}Sb_x$ alloys. *J. Phys. Chem. Solids* **59,** 129–134 (1998).
9. Kulbachinskii, V. A., Kytin, V. G., Kudryashov, A. A. & Tarasov, P. M.

Thermoelectric properties of Bi_2Te_3, Sb_2Te_3 and Bi_2Se_3 single crystals with magnetic impurities. *J. Solid State Chem.* **193,** 47–52 (2012).

10. Fukuyado, J., Narikiyo, K., Akaki, M., Kuwahara, H. & Okuda, T. Thermoelectric properties of the electron-doped perovskites $Sr_{1-x}Ca_xTi_{1-y}Nb_yO_3$. *Phys. Rev. B* **85,** 075112 (2012).

11. Chung, D. *et al.* $CsBi_4Te_6$: A high-performance thermoelectric material for low-temperature applications. *Science* **287,** 1024–1027 (2000).

12. Inohara, T., Okamoto, Y., Yamakawa, Y., Yamakage, A. & Takenaka, K. Large Thermoelectric Power Factor at Low Temperatures in One-Dimensional Telluride Ta_4SiTe_4. *Appl. Phys. Lett.* **110,** 183901 (2017).

13. Takahashi, H. *et al.* Colossal Seebeck effect enhanced by quasi-ballistic phonons dragging massive electrons in $FeSb_2$. *Nature Commun.* **7,** 12732 (2016).

14. Ueno, K. *et al.* Electric-field-induced superconductivity in an insulator. *Nature Mater.* **7,** 855–858 (2008).

15. Lee, Y. *et al.* Phase Diagram of Electrostatically Doped $SrTiO_3$. *Phys. Rev. Lett.* **106,** 136809 (2011).

16. Li, M., Graf, T., Schladt, T. D., Jiang, X. & Parkin, S. S. P. Role of Percolation in the Conductance of Electrolyte-Gated $SrTiO_3$. *Phys. Rev. Lett.* **109,** 196803 (2012).

17. Li, M. *et al.* Suppression of ionic liquid gate-induced metallization of $SrTiO_3$(001) by oxygen. *Nano Lett.* **13,** 4675–4678 (2013).

18. Gallagher, P., Lee, M., Williams, J. R. & Goldhaber-Gordon, D. Gate-tunable superconducting weak link and quantum point contact spectroscopy on a

strontium titanate surface. *Nature Phys.* **10,** 748–752 (2014).

19. Stanwyck, S. W., Gallagher, P., Williams, J. R. & Goldhaber-gordon, D. Universal Conductance Fluctuations in Electrolyte-Gated $SrTiO_3$ Nanostructures. *Appl. Phys. Lett.* **103,** 213504 (2013).

20. Reagor, D. W. & Butko, V. Y. Highly conductive nanolayers on strontium titanate produced by preferential ion-beam etching. *Nature Mater.* **4,** 593–596 (2005).

21. Atesci, H. *et al.* On the formation of a conducting surface channel by ionic liquid gating of an insulator. Preprint at http://arxiv.org/abs/1709.01178 (2017).

22. Wu, C. T. *et al.* Heterojunction of $Fe(Se_{1-x}Te_x)$ superconductor on Nb-doped $SrTiO_3$. *Appl. Phys. Lett.* **96,** 122506 (2010).

23. Zhang, W. *et al.* Interface charge doping effects on superconductivity of single-unit-cell FeSe films on $SrTiO_3$ substrates. *Phys. Rev. B* **89,** 060506(R) (2014).

24. Zhang, H. *et al.* Origin of charge transfer and enhanced electron-phonon coupling in single unit-cell FeSe films on $SrTiO_3$. *Nature Commun.* **8,** 214 (2017).

25. Wang, Q.-Y. *et al.* Interface-induced high-temperature superconductivity in single unit-cell FeSe films on $SrTiO_3$. *Chinese Phys. Lett.* **29,** 037402 (2012).

26. Sun, Y. *et al.* High temperature superconducting FeSe films on $SrTiO_3$ substrates. *Sci. Rep.* **4,** 6040 (2014).

27. Wang, Q. *et al.* Thickness dependence of superconductivity and superconductor-insulator transition in ultrathin FeSe films on $SrTiO_3$ (001) substrate. *2D Mater.* **2,** 044012 (2015).

28. Arsenijević, S. *et al.* Pressure effects on the transport coefficients of $Ba(Fe_{1-x}Co_x)_2As_2$. *Phys. Rev. B* **84,** 075148 (2011).

29. Zhu, Z. W. *et al.* Nernst effect of a new iron-based superconductor $LaO_{1-x}F_xFeAs$. *New J. Phys.* **10,** 063021 (2008).

30. Kondrat, A., Behr, G., Büchner, B. & Hess, C. Unusual Nernst effect and spin density wave precursors in superconducting $LaFeAsO_{1-x}F_x$. *Phys. Rev. B* **83,** 092507 (2011).

31. Wang, G., Endicott, L., Chi, H., Lošt'ák, P. & Uher, C. Tuning the Temperature Domain of Phonon Drag in Thin Films by the Choice of Substrate. *Phys. Rev. Lett.* **111,** 046803 (2013).

32. Suemune, Y. Thermal Conductivity of $BaTiO_3$ and $SrTiO_3$ from 4.5° to 300°K. *J. Phys. Soc. Jpn.* **20,** 174–175 (1965).

33. Liu, D. *et al.* Electronic origin of high-temperature superconductivity in single-layer FeSe superconductor. *Nature Commun.* **3,** 931 (2012).

34. He, S. *et al.* Phase diagram and electronic indication of high-temperature superconductivity at 65 K in single-layer FeSe films. *Nature Mater.* **12,** 605–610 (2013).

35. He, J. *et al.* Electronic Evidence of an Insulator-Superconductor Transition in Single-Layer FeSe/$SrTiO_3$ Films. *Proc. Natl. Acad. Sci. U. S. A.* **111,** 18501–18506 (2014).

36. Miyata, Y., Nakayama, K., Sugawara, K., Sato, T. & Takahashi, T. High-temperature superconductivity in potassium-coated multilayer FeSe thin films. *Nature Mater.* **14,** 775–779 (2015).

37. Ye, Z. R. *et al.* Simultaneous emergence of superconductivity, inter-pocket scattering and nematic fluctuation in potassium-coated FeSe superconductor. Preprint at http://arxiv.org/abs/1512.02526 (2015).

38. Wen, C. H. P. *et al.* Anomalous correlation effects and unique phase diagram of electron-doped FeSe revealed by photoemission spectroscopy. *Nature Commun.* **7,** 10840 (2016).

39. Shi, X. *et al.* Enhanced superconductivity accompanying a Lifshitz transition in electron-doped FeSe monolayer. *Nature Commun.* **8,** 14988 (2017).

40. Uesugi, E., Goto, H., Eguchi, R., Fujiwara, A. & Kubozono, Y. Electric double-layer capacitance between an ionic liquid and few-layer graphene. *Sci. Rep.* **3,** 1595 (2013).

41. Chu, L. *et al.* Charge transport in ion-gated mono-, bi-, and trilayer MoS_2 field effect transistors. *Sci. Rep.* **4,** 7293 (2014).

42. Saito, Y., Kasahara, Y., Ye, J., Iwasa, Y. & Nojima, T. Metallic ground state in an ion-gated two-dimensional superconductor. *Science* **350,** 409–413 (2015).

43. Saito, Y. *et al.* Superconductivity protected by spin–valley locking in ion-gated MoS_2. *Nature Phys.* **12,** 144–149 (2016).

44. Suzuki, Y. *et al.* Momentum-dependent sign inversion of orbital order in superconducting FeSe. *Phys. Rev. B* **92,** 205117 (2015).
